# Iron Excess Stress Promotes Adventitious Root Formation at the Root–Hypocotyl Junction in Arabidopsis

**DOI:** 10.1111/pce.70860

**Published:** 2026-09-08

**Authors:** Poonam Kanwar, Britta Möllmann, Stan Altmeisch, Petra Bauer

**Affiliations:** ^1^ Institute of Botany Heinrich Heine University Düsseldorf Germany; ^2^ Cluster of Excellence on Plant Science (CEPLAS) Heinrich Heine University Düsseldorf Germany

## Abstract

Fe excess triggers auxin‐mediated adventitious root formation at the root–hypocotyl junction (J‐ARs), helping Arabidopsis maintain root growth when primary root function is compromised under Fe toxicity.

Plants adapt to environmental stress through developmental plasticity. Although iron (Fe) is essential for growth, excessive Fe can become toxic, particularly under waterlogged or acidic conditions that enhance Fe^2+^ availability. In this study, Arabidopsis seedlings overexpressing the Fe uptake regulator bHLH039 (39Ox), which enhances the FIT‐mediated Fe uptake pathway, were used to study root responses to excess Fe (Naranjo‐Arcos et al. 2017; Kanwar et al. 2025). A time‐course analysis of 39Ox seedlings revealed a progressive increase in adventitious root formation at the root–hypocotyl junction (J‐ARs) from 3 to 14 days (Figure 1A). At 6 days, primary root elongation was inhibited and J‐ARs initiated at the root–hypocotyl junction. By 10 and 14 days, most seedlings developed one or more J‐ARs (Figures 1A and S1). J‐AR formation occurred in ~12% of WT seedlings but increased to 79% in 39Ox seedlings. In contrast, J‐AR formation in 39Ox was reduced to 5% under –Fe conditions (Figure 1B), indicating that Fe excess is a key trigger of this developmental response. As previously reported, FIT overexpression does not constitutively activate Fe uptake (Colangelo and Guerinot 2004). FIT‐overexpressing seedlings showed WT‐like Fe staining and no J‐AR formation, indicating that 39Ox J‐AR development is likely due to Fe toxicity rather than other bHLH effects (Figure S2).

**Figure 1:**
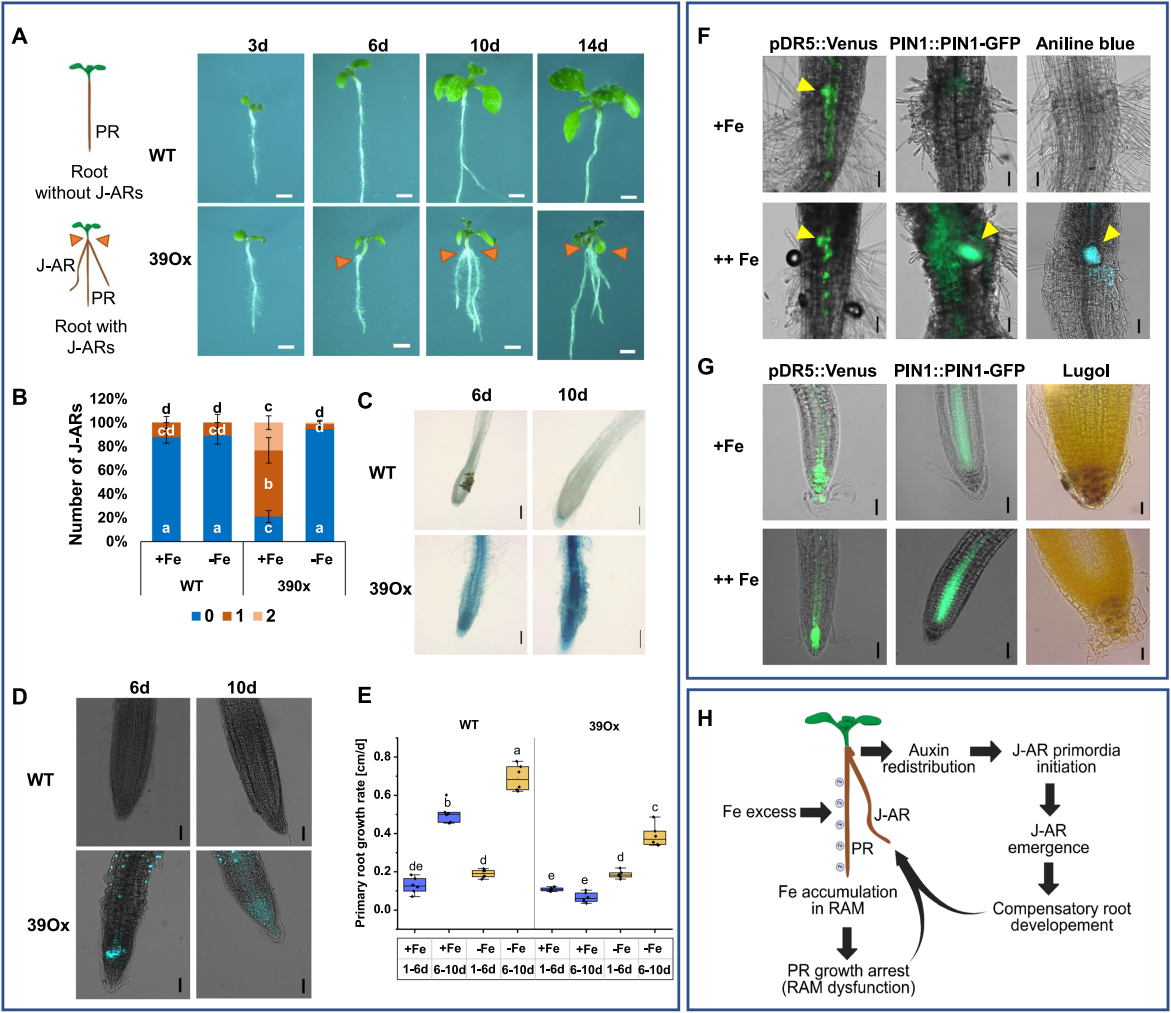
Iron (Fe) excess stress induces adventitious root formation at the root–hypocotyl junction (J‐ARs) in Arabidopsis. (A) Time‐course analysis of root architecture in WT and 39Ox seedlings grown vertically for 14 days. J‐AR emergence is shown at 3‐, 6‐, 10‐, and 14‐day‐old stages. Scale bar, 1000 μm. (B) WT and 39Ox seedlings grown under +Fe and −Fe conditions for 10 days. J‐AR numbers are quantified as the percentage of seedlings with zero, one, or two J‐ARs. Bars represent mean ± SDM (*n* = 3; 30 seedlings per experiment). (C) Perls staining in root tips of 6 and 10 days seedlings. Scale bar, 100 μm. (D) Callose deposition detected by aniline blue staining in root tips of 6 and 10 days seedlings under +Fe conditions. Scale bar, 50 μm. (E) Growth rate, calculated as total root length divided by number of days, shown in the bottom panel. For each treatment, the average of 10 seedlings was used for calculation. Bars represent mean ± SD (*n* = 6). (F) Localisation of pDR5::3xVenus‐N7 (6 days), PIN1:PIN1‐GFP (5 days), and aniline blue staining (6 days) at the root–shoot junction in WT seedlings grown under +Fe and ++Fe conditions. Scale bar, 50 μm. (G) Localisation of pDR5::3xVenus‐N7 and PIN1:PIN1‐GFP (Scale bar, 50 μm), together with Lugol staining (Scale bar, 10 µm), at the root tip in 6 days WT seedlings grown under +Fe and ++Fe conditions. (H) Schematic model illustrating regulation of J‐AR formation under Fe toxicity conditions. The figure was prepared using BioRender (https://BioRender.com). For panels (B) and (E), different letters indicate statistically significant differences between samples (ANOVA followed by Tukey's test, *p* < 0.05). PR: primary root, RAM: root apical meristem. Orange and yellow triangles denote J‐ARs and J‐AR primordia, respectively.

Ammonium (NH_4_^+^) predominates in anaerobic, waterlogged soils and is associated with Fe bioavailability (Coleto et al. 2023). NH_4_^+^ supplementation inhibited primary root growth and enhanced J‐AR formation in 39Ox, increasing both the frequency of J‐ARs and seedlings with two J‐ARs (Figure S3A–C). Notably, NH_4_^+^ supplementation did not induce ferric reductase activity under Fe‐deficient conditions (Figure S3D). NH_4_^+^‐induced rhizosphere acidification can increase Fe solubility and availability (Britto and Kronzucker 2002), thereby suppressing Fe deficiency responses and promoting J‐AR development in 39Ox seedlings.

The root tip is the critical site in the arrest of Arabidopsis primary root growth under Fe toxicity (Zhang et al. 2018). To examine the link between RAM dynamics and J‐AR development, we analysed 6‐ and 10‐day‐old seedlings. Strong Fe accumulation was detected in the RAM of 39Ox roots at both stages, with greater staining at 10 days, whereas WT showed little or no staining (Figure 1C). Excess Fe in the root meristem induces callose deposition in the root tip and modulates meristem activity (Müller et al. 2015). Accordingly, 39Ox seedlings accumulated callose in the root tip, unlike WT seedlings, where it was undetectable in the RAM (Figure 1D). To examine the relationship between primary root growth and J‐AR formation, we compared growth rates in WT and 39Ox seedlings before (0–6 days) and after (6–10 days) J‐AR emergence (Figure 1E). Growth rate increased in WT under both Fe conditions and 39Ox under –Fe, but not in 39Ox under +Fe. Thus, Fe‐induced primary root inhibition in 39Ox coincides with J‐AR initiation, suggesting a stress‐adaptive response. Further, Turnbull staining showed minimal Fe^2+^ accumulation in 6‐ and 10‐day‐old WT primary roots under +Fe conditions, whereas 39Ox roots showed strong Fe accumulation, particularly at 10 days (Figure S4). In contrast, J‐ARs of 39Ox plants showed minimal or undetectable Fe staining, likely due to their younger developmental age and shorter Fe exposure. Thus, J‐ARs may function as temporary roots, sustaining growth under Fe toxicity by providing a favourable environment when the primary root is compromised.

WT seedlings grown under Fe‐excess conditions also developed J‐ARs, showing that elevated Fe is sufficient to trigger this response (Figure S5). Because root architecture is regulated by auxin distribution, we examined auxin dynamics during J‐AR formation (Li et al. 2015). pDR5::3×Venus‐N7 expression in J‐AR primordia under both control and Fe‐excess conditions indicates that auxin response occurs independently of Fe availability and is sufficient for primordium initiation (Figures 1F and S6). In contrast, PIN1 expression and enhanced callose deposition were observed only under Fe toxicity (Figures 1F and S7). Given the roles of PIN‐dependent auxin transport in organ formation and callose‐mediated restriction of symplastic connectivity, these observations suggest that excess Fe promotes mechanisms reinforcing auxin canalisation and local auxin retention essential for subsequent outgrowth of J‐AR primordia (Benková et al. 2003).

In the root meristem, PIN1 localisation remained unchanged under Fe toxicity, while DR5 reporter activity under Fe‐excess conditions was restricted to the stem cell niche, suggesting that excess Fe disrupts auxin distribution by limiting both lateral and distal auxin diffusion (Figure 1G). Since auxin positively regulates amyloplast biogenesis in the root meristem, Lugol staining was used to visualise starch‐filled amyloplasts in columella cells. Fe excess reduced starch accumulation in WT roots (Figure 1G) and caused an almost complete loss in 39Ox seedlings (Figure S8). Transcriptome analysis of 6‐day‐old 39Ox and WT seedlings (Naranjo‐Arcos et al. 2017), identified differential expression of several auxin‐related genes (Table S1; Figure S9). These transcriptional changes were largely attenuated under Fe‐deficient conditions, where J‐AR formation is absent (Figure S10). In summary, Fe overload triggers an auxin‐mediated developmental response that remodels root architecture by promoting J‐AR formation when primary root function is compromised (Figure 1H).

## Conflicts of Interest

The authors declare no conflicts of interest.

## Supporting information


Supporting File 1

Supporting File 2

Supporting File 3

## Data Availability

The microarray datasets are publicly available in CATdb (AU13‐07_Ox) and GEO (GSE93093). Additional information and materials are available from the corresponding author upon reasonable request.
