# Supplementary material for: Iron Excess Stress Promotes Adventitious Root Formation at the Root–Hypocotyl Junction in Arabidopsis
Source: Plant Cell Environ. 2026 Sep 8;49(12):10061–3. doi: 10.1111/pce.70860 (PMC13725250; doi:10.1111/pce.70860)
Supplement: Supplementary file 1 [file PCE-49-10061-s002.pdf]

Fig S1

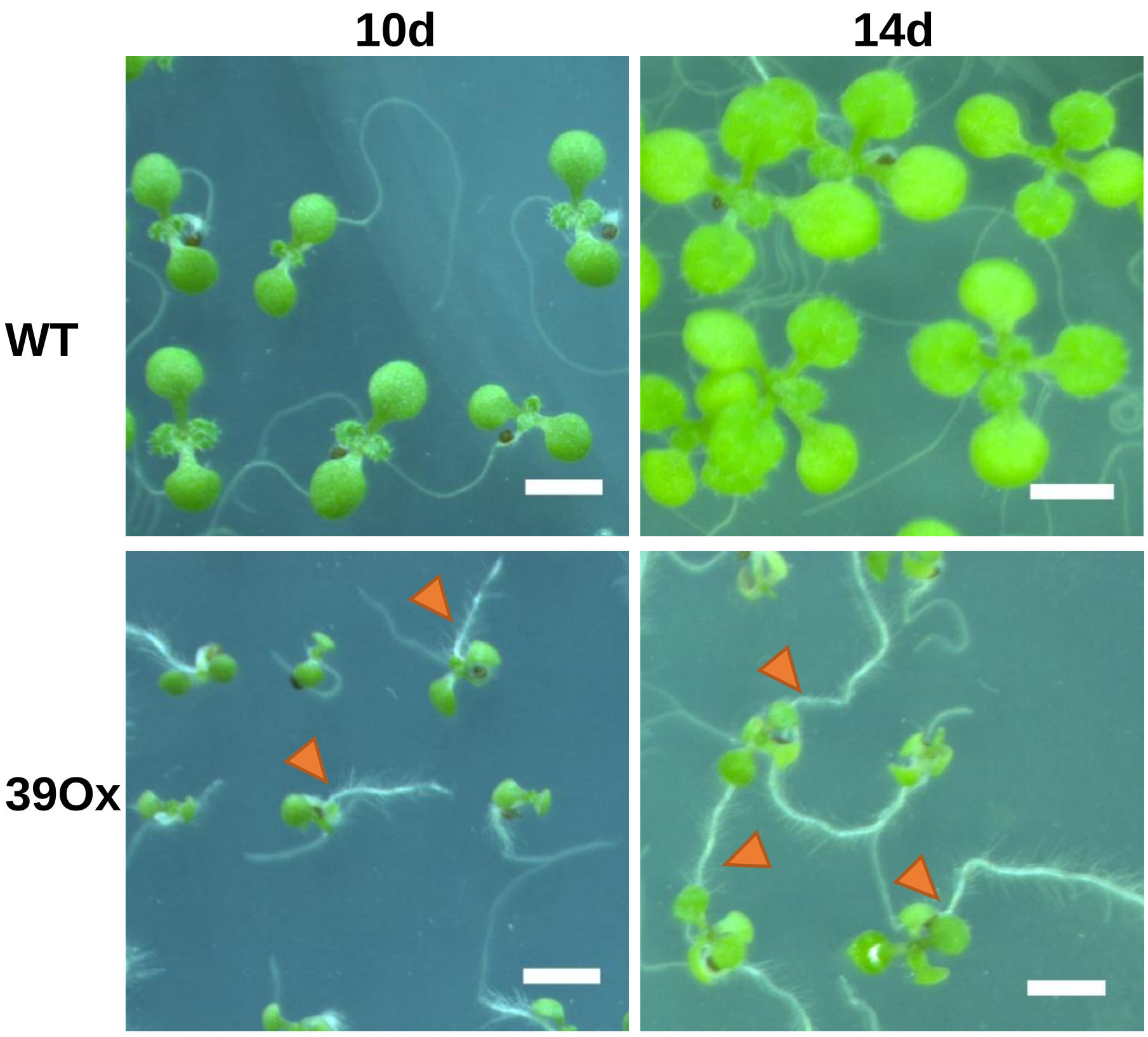

Figure S1: J-AR emergence at the surface of the medium in 39Ox seedlings. WT and 39Ox seedlings were grown for 10 and 14 days in plates placed horizontally. Scale bar, 2000 μm. At 10 days, J-ARs began to emerge at the surface of the medium in 39Ox seedlings. By 14 days, a higher number of J-ARs was observed at the surface. Orange triangles indicate anchor roots. d: days.

Fig S2

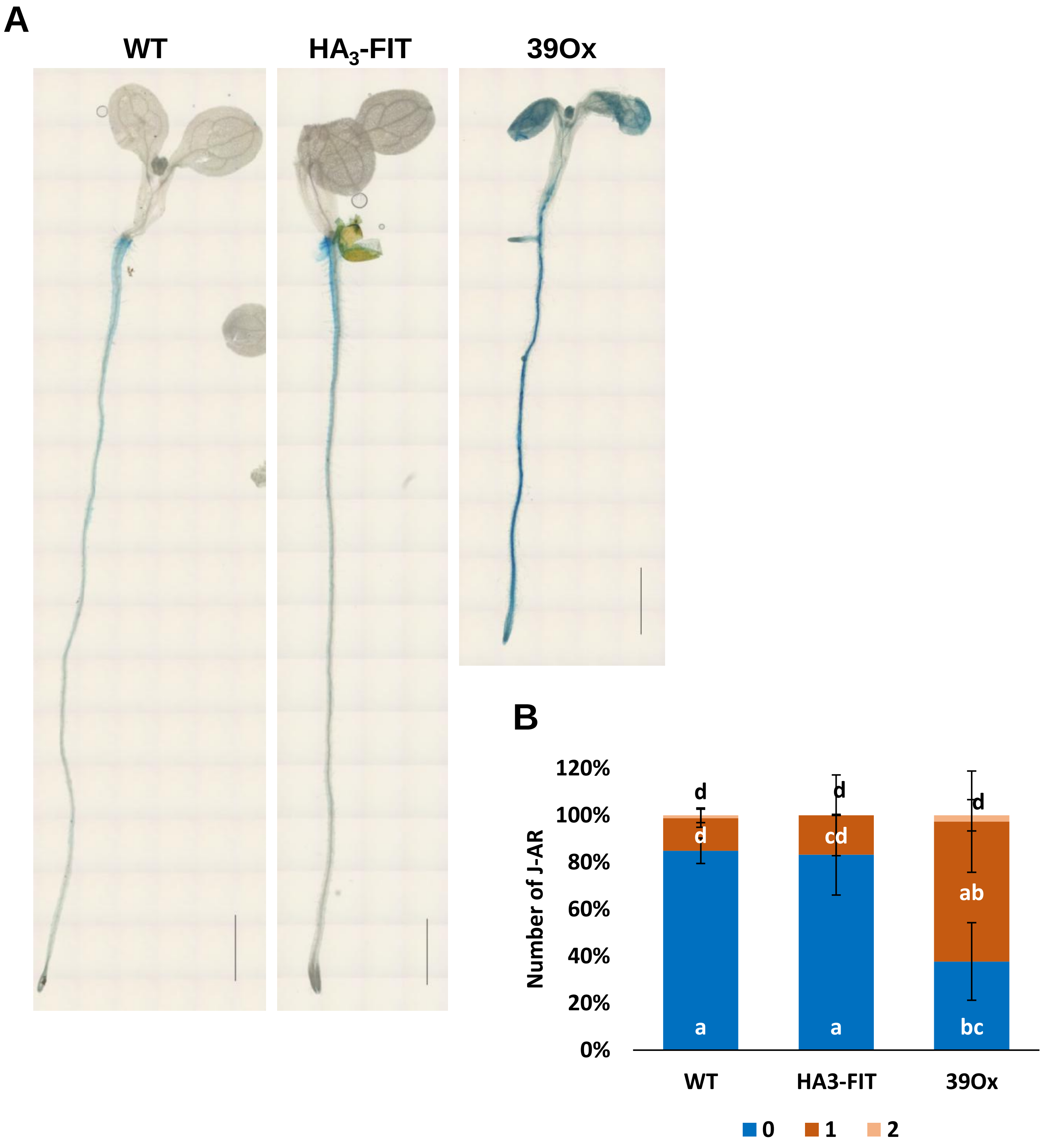

Figure S2: Perl staining and quantification of J-AR number in WT, HA_3-FIT, and 39Ox seedlings. (A) Perl staining of WT, HA_3-FIT, and 39Ox 6-day-old seedlings under +Fe conditions (n=5; scale bar=0.1 cm). (B) seedlings were grown under +Fe, conditions for 10 days. Quantification of J-AR number as a percentage of seedlings with zero, one, or two J-AR corresponding to the genotype in the bottom panel. Bars represent mean ± SDM (n = 6; 20 seedlings per experiment were analyzed).

Fig S3

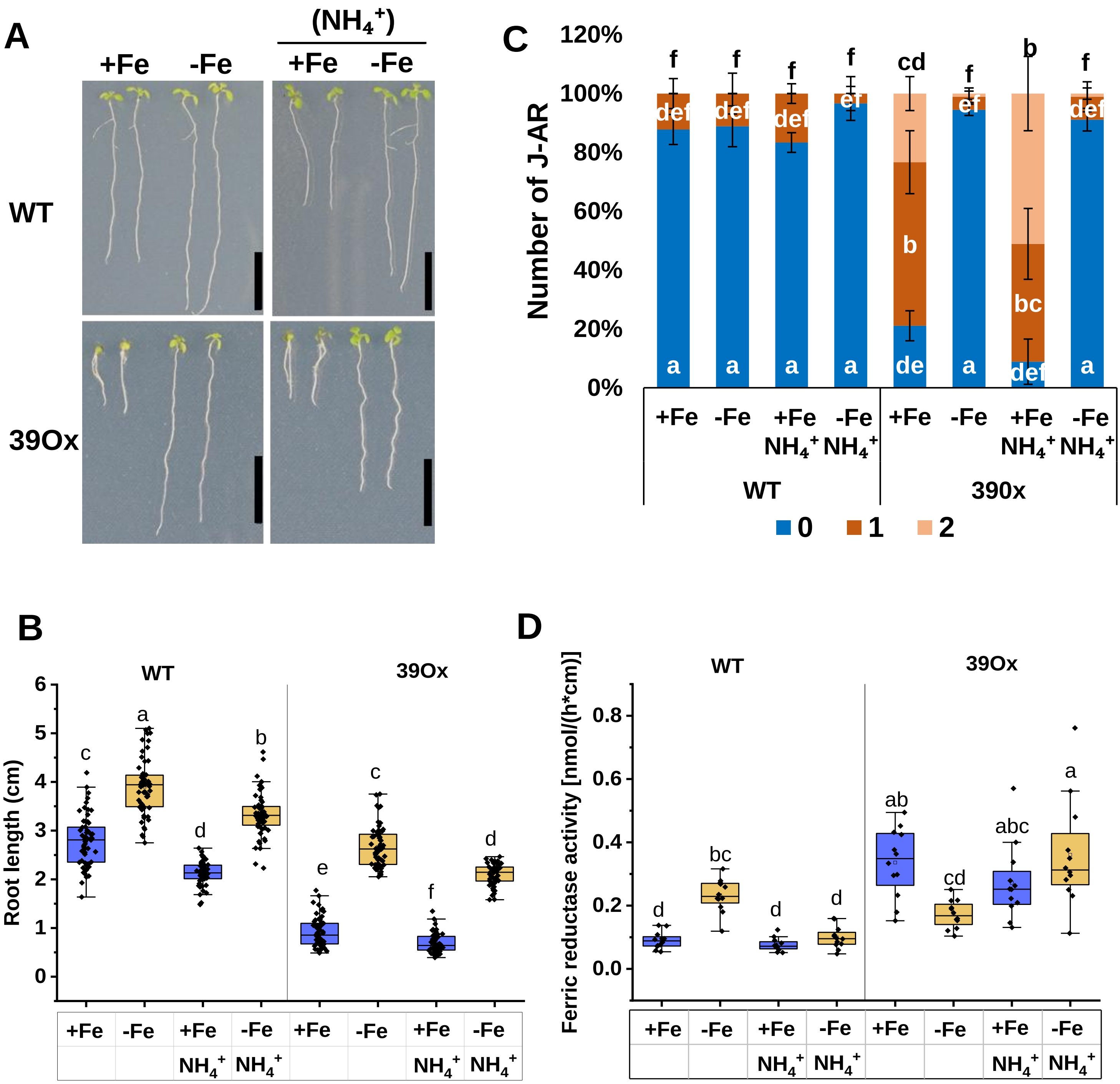

Figure S3: Supplementation with ammonium (NH_4^+) enhanced J-AR formation in 39Ox. 39Ox and WT seedlings were grown under +Fe or −Fe media with and without NH_4^+ supplementation for 10 days. (A) Whole seedling growth of a ten-day-old seedling (Scale bar, 1 cm). (B) Root lengths of ten-day-old seedlings (n = 60; seedlings from 3 independent experiments). (C) Quantification of J-AR no. in media as a percentage of ten-day-old seedlings with zero, one, or two J-ARs corresponding to the treatments in the bottom panel. Bars represent mean ± SDM (n = 3; 30 seedlings per experiment were analyzed). (D) Root Fe reductase activity in ten-day-old seedlings (n = 12; seedlings from 3 independent experiments). Bars represent mean ± SDM. Different letters indicate statistically significant differences (ANOVA and Tukey test, $p < 0.05$).

Fig S4

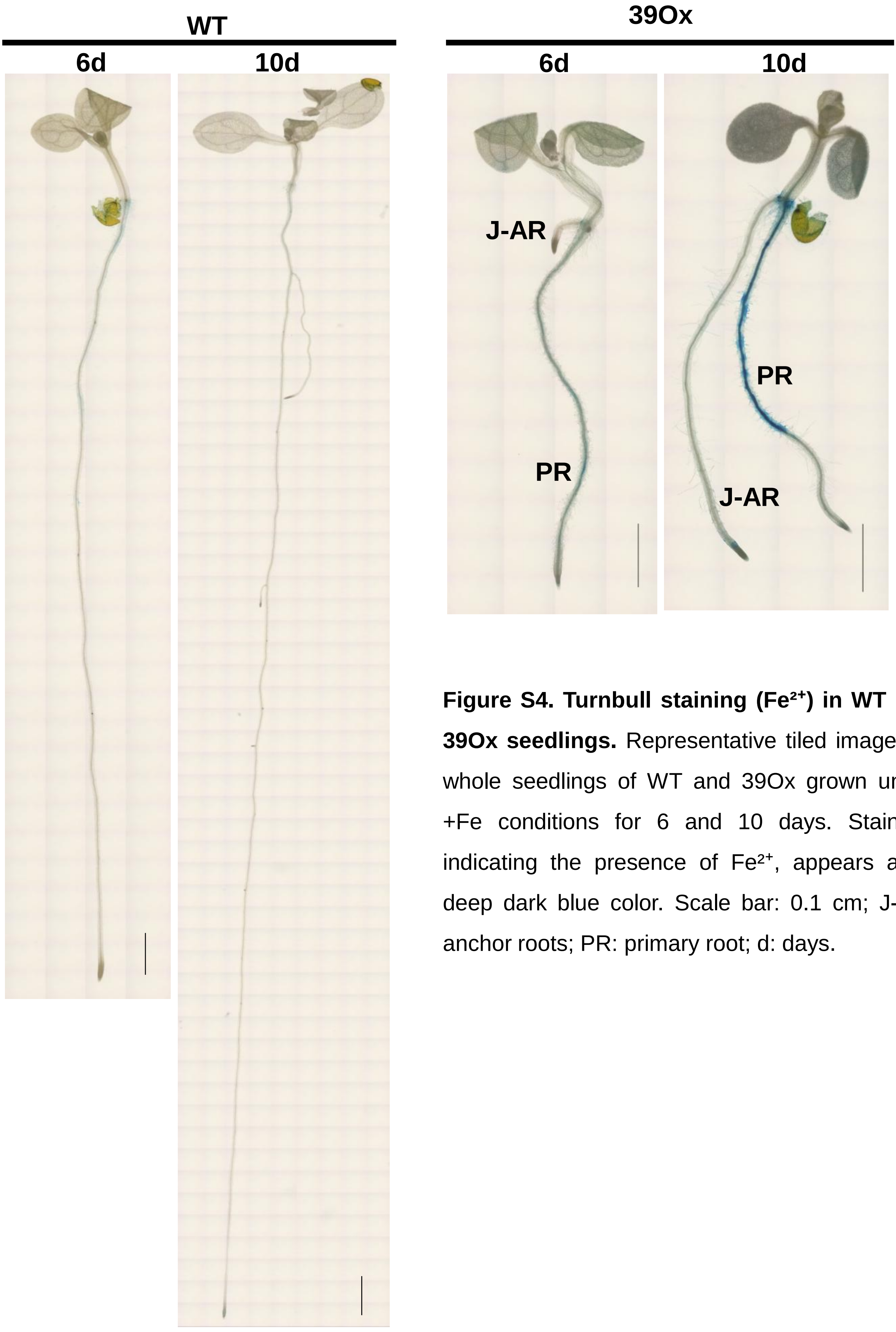

Figure S4. Turnbull staining (Fe^{2+}) in WT and 39Ox seedlings. Representative tiled images of whole seedlings of WT and 39Ox grown under +Fe conditions for 6 and 10 days. Staining, indicating the presence of Fe^{2+}, appears as a deep dark blue color. Scale bar: 0.1 cm; J-AR: anchor roots; PR: primary root; d: days.

Fig S5

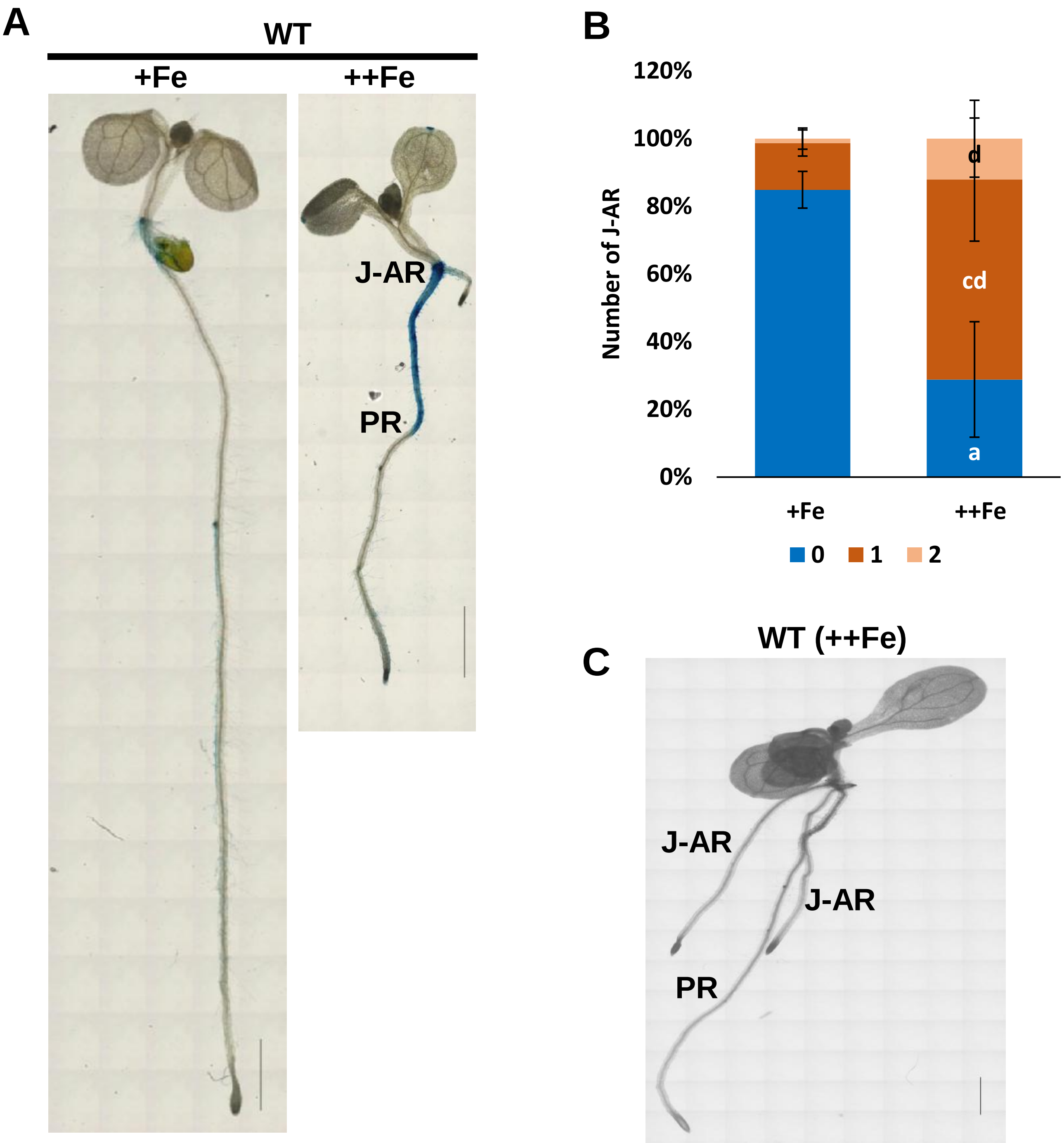

Figure S5: Perl staining and quantification of J-AR number in WT seedlings under +Fe and ++Fe conditions. (A) Perl staining of WT 6-day-old seedlings under +Fe and ++Fe conditions (n = 5; scale bar=0.1 cm). The emergence of J-ARs can be observed under ++Fe conditions. (B) WT seedlings were grown under +Fe and ++Fe conditions for 10 days. Quantification of J-AR number as a percentage of seedlings with zero, one, or two J-AR corresponding to the treatments in the bottom panel. Bars represent mean ± SDM (n = 6; 20 seedlings per experiment were analyzed). (C) Image of 10-day-old WT seedlings with two J-ARs. (Scale bar=0.1 cm)

Fig S6

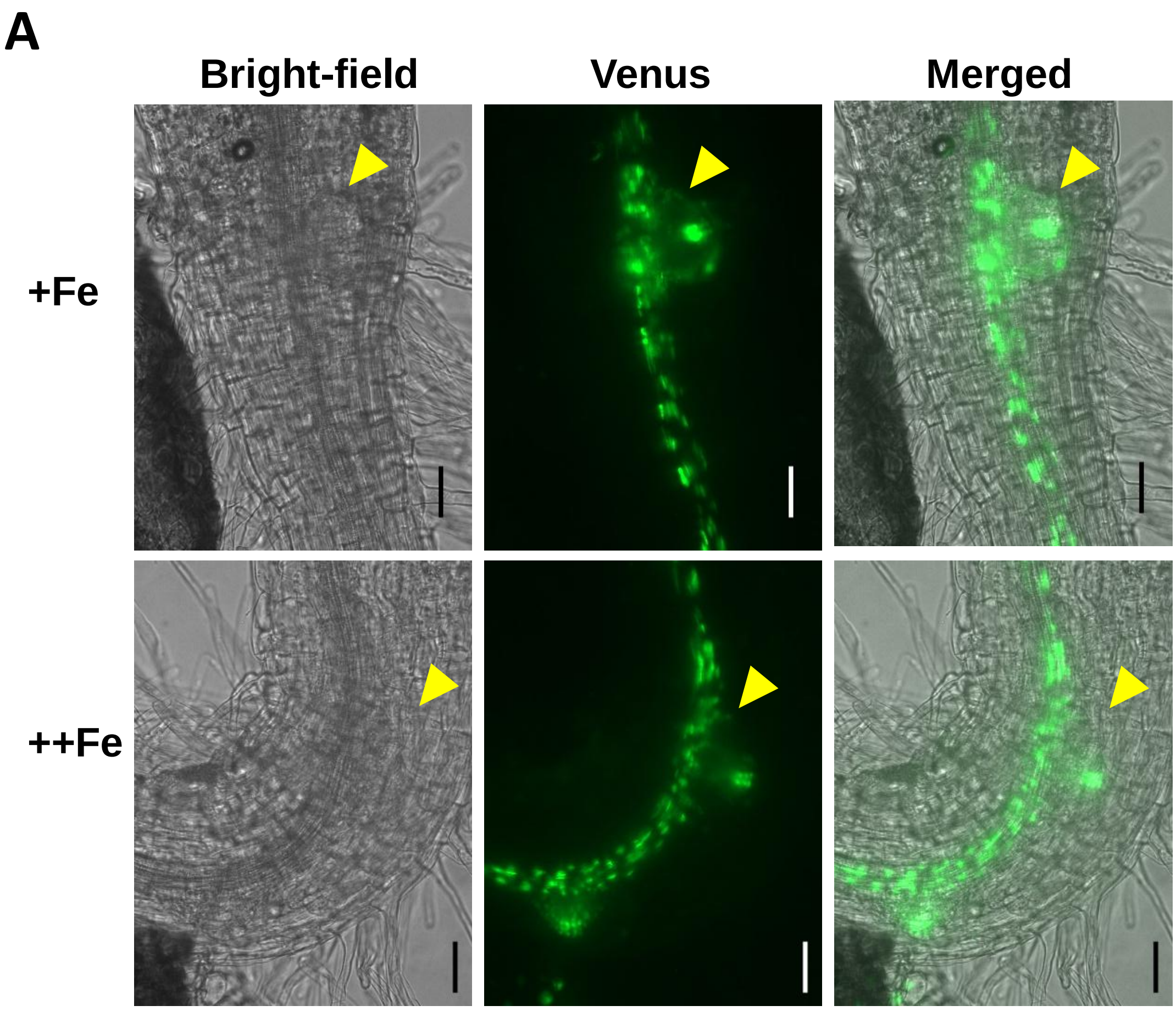

Figure S6. Localization of pDR5::3xVenus-N7 at the root–shoot junction in WT seedlings grown under +Fe and ++Fe conditions. Representative images show the spatial distribution of pDR5::3×Venus-N7 fluorescence in J-AR primordia at the root–shoot junction region of WT seedlings under both +Fe and ++Fe conditions. Yellow triangles indicate the position of J-AR primordia. Scale bar = 50 µm.

Fig S7

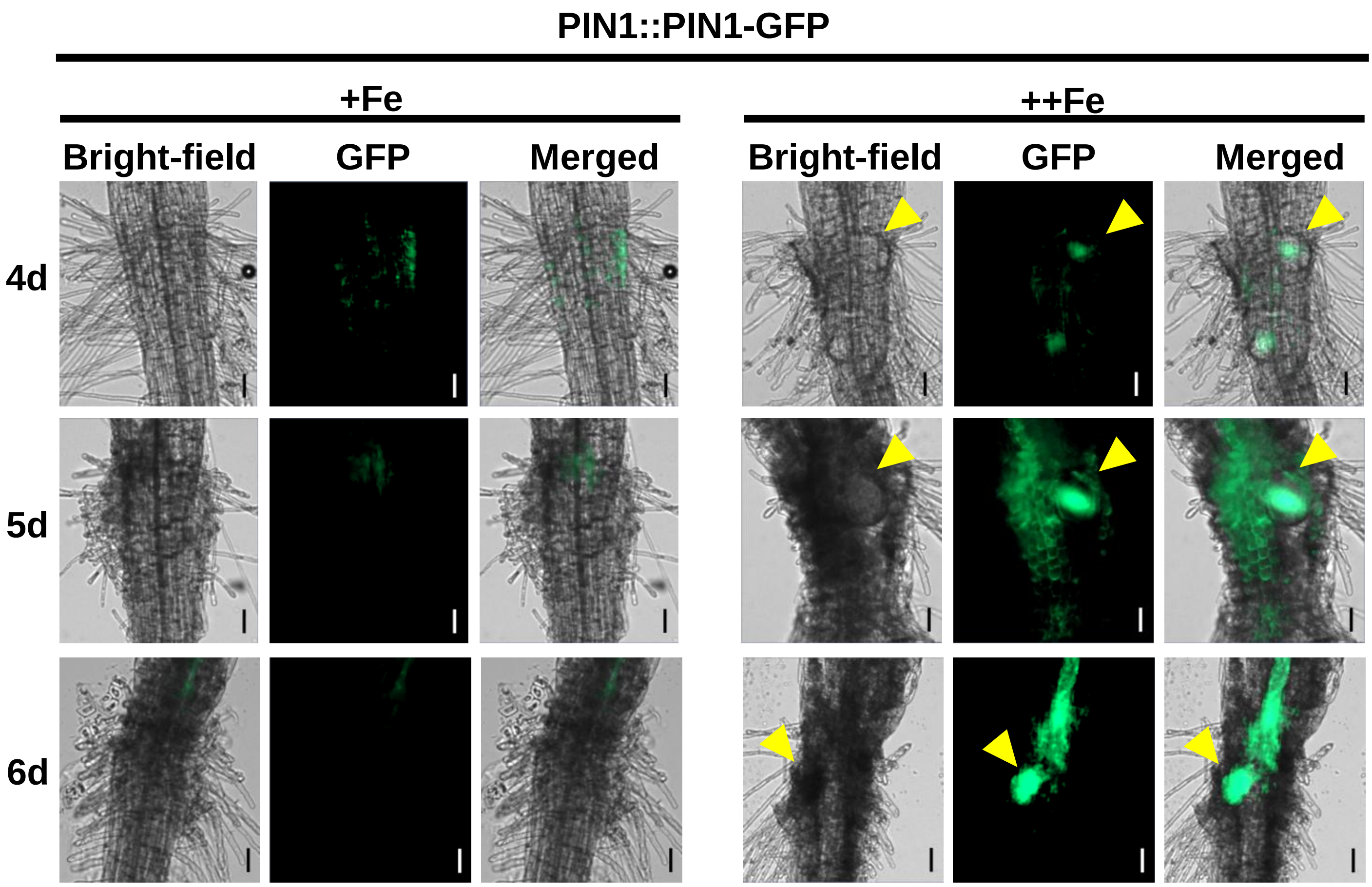

Figure S7. Localization of PIN1:PIN1-GFP at the root–shoot junction of WT seedlings grown under +Fe and ++Fe conditions. Representative images show the spatial distribution of PIN1 fluorescence in the root–shoot junction region of 4-, 5-, and 6-day-old seedlings. Under ++Fe conditions, PIN1 fluorescence strongly accumulates in the root–shoot junction and developing J-AR primordia, with signal intensity progressively increasing from 4 to 6 days. In contrast, PIN1 fluorescence is weak or undetectable under +Fe conditions. Yellow triangles indicate the position of J-AR primordia. Scale bar = 50 µm.

Fig S8

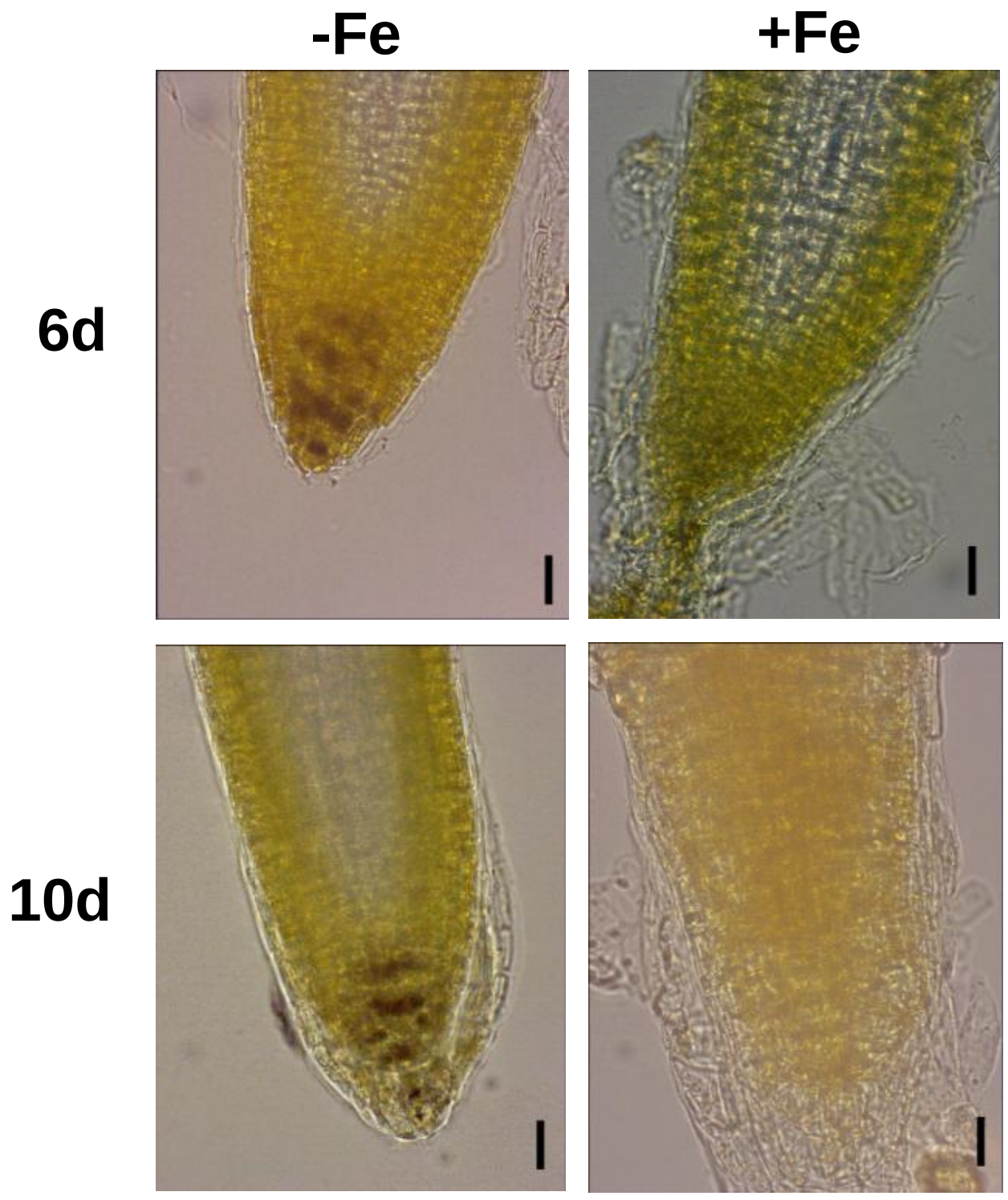

Figure S8. Lugol staining in 39Ox seedlings. Representative images of Lugol staining at the root tip of 39Ox seedlings grown under +Fe or −Fe conditions for 6 and 10 days. Scale bar, 10 μm.

Fig S9

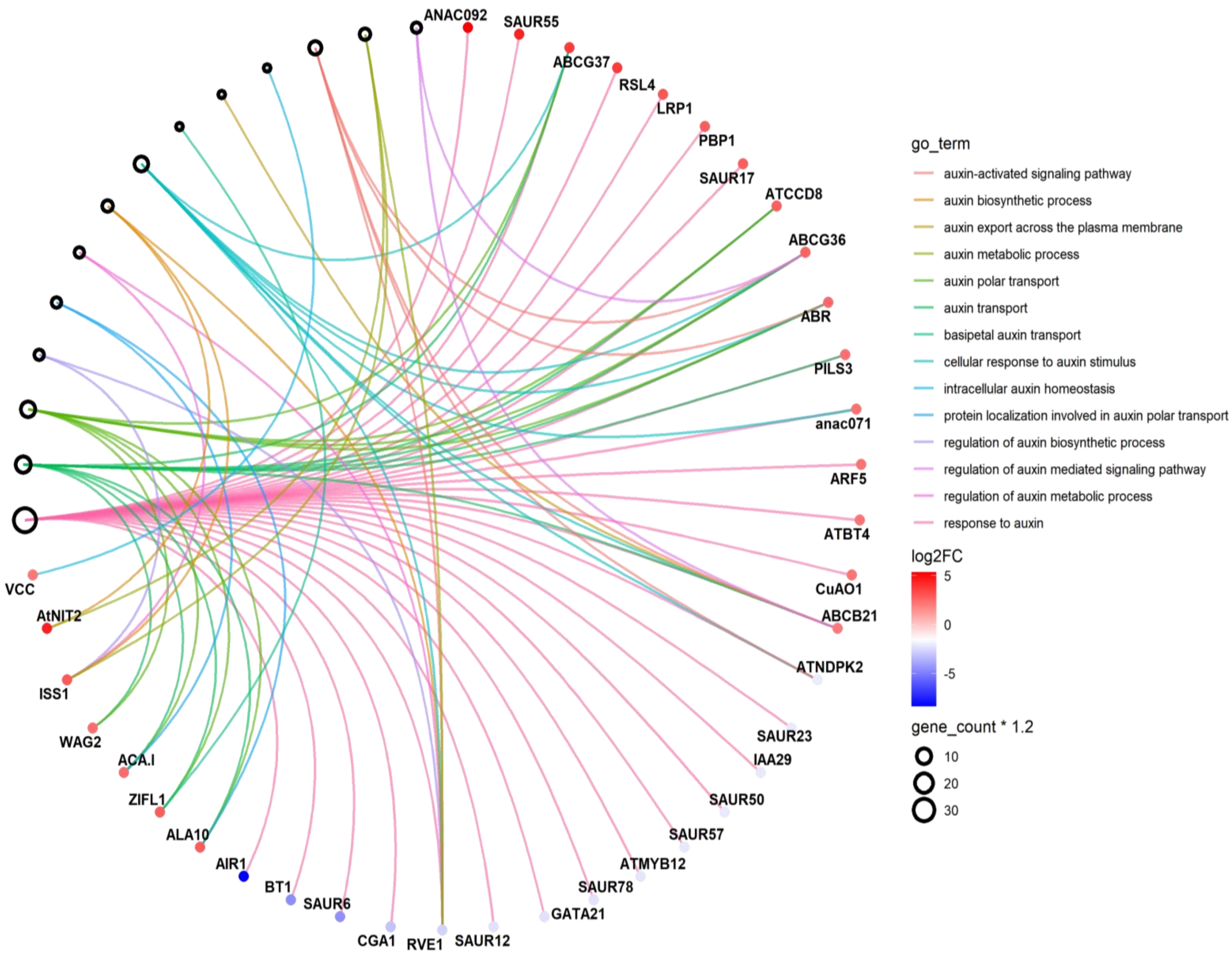

Figure S9. Auxin-related transcriptional changes during J-AR initiation in 39Ox seedlings under +Fe conditions. Circular chord diagram showing auxin-related Gene Ontology (GO) enrichment analysis of differentially expressed genes (DEGs) with ≥2-fold expression changes in 39Ox compared to WT under +Fe conditions in 6-day-old seedlings. Each colored curve connects DEGs to their corresponding GO terms (colored by functional category). The color gradient represents the $\log_2$ fold change (logFC) in expression, with red indicating upregulation and blue indicating downregulation relative to WT.

Fig S10

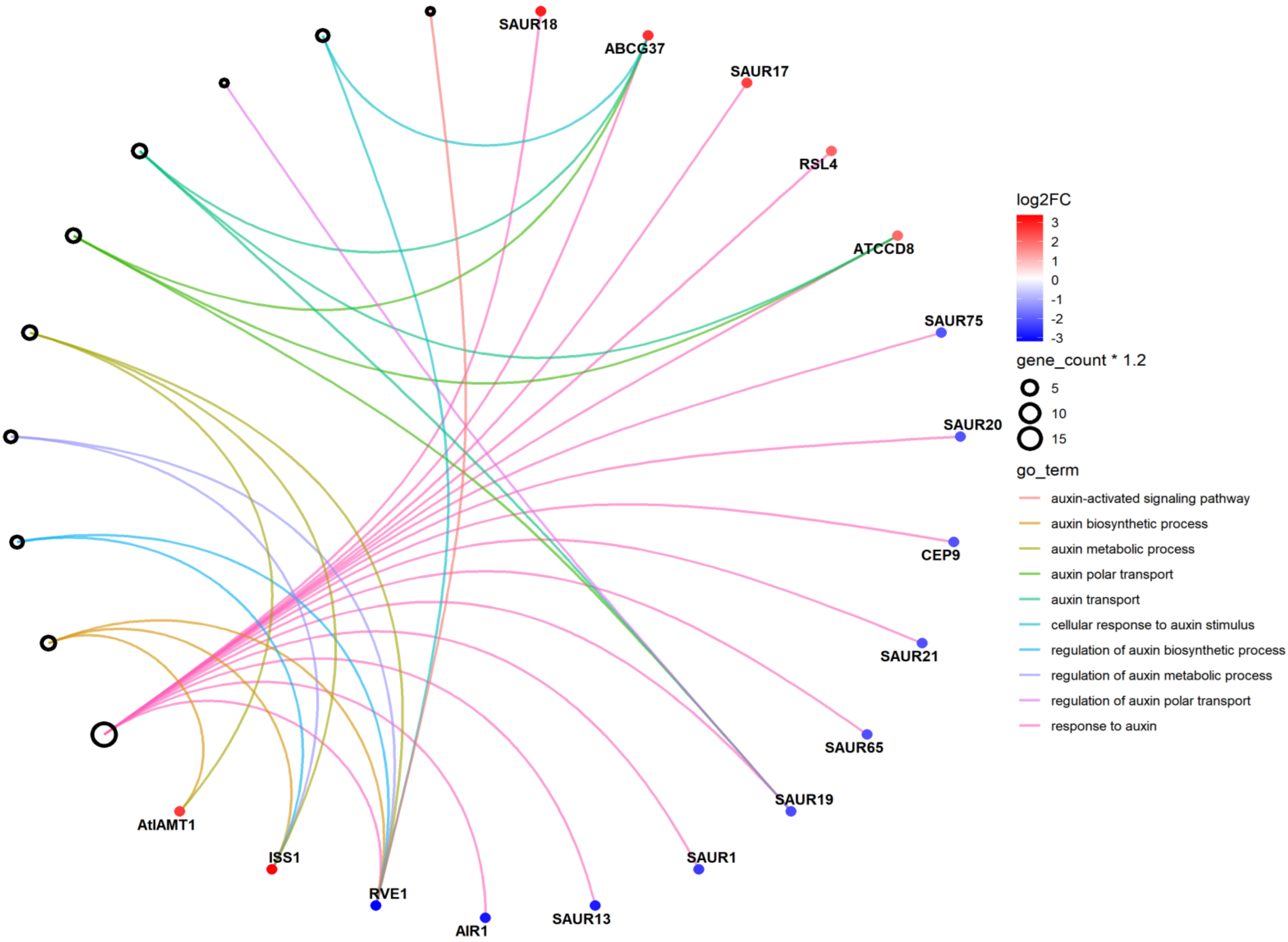

Figure S10. Auxin-related transcriptional changes during J-AR initiation in 39Ox seedlings under -Fe conditions. Circular chord diagram showing auxin-related Gene Ontology (GO) enrichment analysis of differentially expressed genes (DEGs) with ≥2-fold expression changes in 39Ox compared to WT under -Fe conditions in 6-day-old seedlings. Each colored curve connects DEGs to their corresponding GO terms (colored by functional category). The color gradient represents the $\log_2$ fold change (logFC) in expression, with red indicating upregulation and blue indicating downregulation relative to WT.
