# Supplementary material for: Iron Excess Stress Promotes Adventitious Root Formation at the Root–Hypocotyl Junction in Arabidopsis
Source: Plant Cell Environ. 2026 Sep 8;49(12):10061–3. doi: 10.1111/pce.70860 (PMC13725250; doi:10.1111/pce.70860)
Supplement: Supplementary file 2 [file PCE-49-10061-s001.docx]

**Materials and methods**

**Plant material and growth media**

Wild-type Arabidopsis ecotype Columbia-0 (WT), bHLH039 overexpression (39Ox) lines (Naranjo-Arcos *et al.* 2017), HA3-FIT (HA-FIT9) line (Meiser, Lingam & Bauer 2011), pDR5::3x Venus-N7 line (Heisler *et al.* 2005), and PIN1:PIN1-GFP (Benková *et al.* 2003) were used in this study. Modified half-strength Hoagland agar medium was prepared as previously described (Kanwar, Altmeisch & Bauer 2025). Modified half-strength Hoagland agar medium was prepared as previously described (Kanwar, Altmeisch & Bauer 2025). The medium contained 1.5 mM Ca(NO₃)₂, 1.25 mM KNO₃, 0.75 mM MgSO₄, 0.5 mM KH₂PO₄, 50 μM KCl, 50 μM H₃BO₃, 10 μM MnSO₄, 2 μM ZnSO₄, 1.5 μM CuSO₄, 0.075 μM (NH₄)₆Mo₇O₂₄, 1% (w/v) sucrose, and 1.4% (w/v) plant agar (Duchefa), adjusted to pH 5.8. Iron was supplied as Fe(III)NaEDTA at 50 μM for iron-sufficient conditions (+Fe), omitted for iron-deficient conditions (−Fe), and increased to 150 μM for iron-excess conditions (++Fe). For ammonium supplementation, KH₂PO₄ was replaced with an equimolar concentration of NH₄H₂PO₄.

**Plant growth conditions**

Arabidopsis seeds were surface sterilized with sterilisation solution (6% sodium hypochlorite (NaOCl) and 0.1% TritonX) for 8 min and washed five times with distilled water. Seeds were stratified for 2d in 0.1% plant agar in the dark at 4°C. For vertical growth, seeds were plated in two rows of 20 seeds, and for horizontal growth, seeds were plated at equal distances on Hoagland agar medium containing different iron (Fe) conditions in square Petri dishes. The plates were then placed in a growth chamber with temperature of 21°C and a 16-hour light cycle from 6 am to 10 pm. The seeds were allowed to germinate and grow in square plates under different conditions for 6d, 10d, and 14d.

**Root phenotype**

For root phenotype WT and 39Ox plants were grown on agar vertically and horizontally in square plates till 14 days. Plates were photographed at different time points. Plates were photographed at different time points using a stereo zoom microscope (Axio Zoom.V16 Stereo-Zoom-Microscope (Zeiss, Jena, Germany).

**J-ARs number, root length, and ferric reductase activity**

WT and 39Ox plants were grown vertically in square plates for 10d and plates were photographed. The photographs were used to calculate the J-ARs per seedling and the root length. Root length was measured from frontal images of plant roots using the JMicroVision software (http://www.jmicrovision.com) as described in (Ivanov *et al.* 2014). Iron reductase activity was determined using a quantitative liquid assay with ferrozine as indicator for Fe^2+^. The activity of the root surface ferric reductase was measured spectrophotometrically as described in (Le *et al.* 2016) and normalized to root length.

**Seedling fixation**

WT and 39Ox plants were grown vertically in square plates for 6 and 10d. Seedlings were placed in 1 ml of fixation solution (Methanol: Chloroform: Acetic acid 6:3:1) for 5 minutes. The solution was then removed and replaced with another 1 ml of fixative. The seedlings were placed in this solution at 4°C for 24 hours. After incubation, the solution was removed, and the seedlings were washed three times with H_2_O before being stored in 70% Ethanol.

**Histochemical detection of iron**

To localize Fe^3+^, fixed seedlings were vacuum infiltrated with pre-warmed (37°C) Perl's stain solution (equal volumes of 4% [v/v] HCl and 4% [w/v] K-ferrocyanide) for 30 min. The stain solution was removed, and the seedlings were washed three times with water. To localize Fe^2+^, the fixed seedlings were incubated with Turnbull's stain solution (Potassium Ferricyanide – 0.4gm and HCl 0.006N – 40ml) for 1h at RT. The stain solution was removed, and the seedlings were washed with 1% acetic acid. Rinse seedlings three times with water. Light microscopy (LM,400-700nm range), was used for ferric and ferrous iron localisation. For this purpose, the roots were imaged on an AxioImager. M2 microscope (Zeiss). Images were taken under Brightfield using a Plan-Apochromat 10x objective and recorded by an Axiocam 105 camera. “Tiles” module and the “stitching” function of the software ZEN 2 (Zeiss) to assemble the collected images.

**Aniline blue staining**

Fixed WT and 39Ox seedlings were washed in 150 mM K₂HPO₄ for 30 min and subsequently incubated in staining solution containing 150 mM K₂HPO₄ and 0.01% (w/v) aniline blue for 2 h in the dark (Schenk & Schikora 2015). Samples were imaged using a Zeiss Axio Imager M2 microscope equipped with an Axiocam 305 camera at 10× magnification and controlled with ZEN software. Aniline blue fluorescence was visualized using a DAPI filter set, and corresponding brightfield images were acquired.

**DR5 and PIN1 localization**

The spatial distribution of auxin response and PIN1 protein was analyzed using the pDR5::3xVenus-N7 and pPIN1::PIN1-GFP reporter lines, respectively. Freshly harvested seedlings were imaged with a Zeiss Axio Imager M2 microscope equipped with an Axiocam 305 camera at 10× magnification using ZEN software. Venus fluorescence was detected using a YFP filter set, and GFP fluorescence was detected using a GFP filter set. Corresponding brightfield images were acquired for all samples.

**Lugol staining**

Starch accumulation was visualized by staining fixed WT and 39Ox seedlings with Lugol’s iodine solution. Stained samples were mounted on microscope slides and imaged using a Zeiss Axio Imager M2 microscope equipped with an Axiocam 105 color camera at 40× magnification. Brightfield images were acquired using ZEN software.

**Auxin-Related Gene Ontology (GO) Enrichment Analysis**

Differentially expressed genes (DEGs) between 39Ox and WT seedlings grown under +Fe and -Fe conditions were identified from microarray data (Naranjo-Arcos et al., 2017). Genes with ≥2-fold change were used for auxin-related GO enrichment (Supplementary Table 1). Enrichment of GO Biological Processes was performed in R using clusterProfiler (v4.10+) with the appropriate *org.*.eg.db annotation. Results (FDR ≤ 0.05) were visualized as chord diagrams using GOplot to illustrate relationships between DEGs and enriched GO terms.

**Statistical analysis**

Three technical repeats and three biological repeats were used. Statistical data analysis was performed using one-way ANOVA followed by a Tukey test in OriginLab software. Graphs were in using OriginLab software. Different lowercase letters in bar graphs indicate statistically significant differences (P < 0.05).

**References**

Benková E., Michniewicz M., Sauer M., Teichmann T., Seifertová D., Jürgens G. & Friml J. (2003) Local, Efflux-Dependent Auxin Gradients as a Common Module for Plant Organ Formation. *Cell* **115**.

Heisler M.G., Ohno C., Das P., Sieber P., Reddy G. V., Long J.A. & Meyerowitz E.M. (2005) Patterns of auxin transport and gene expression during primordium development revealed by live imaging of the Arabidopsis inflorescence meristem. *Current Biology* **15**.

Ivanov R., Brumbarova T., Blum A., Jantke A.M., Fink-Straube C. & Bauer P. (2014) SORTING NEXIN1 Is Required for Modulating the Trafficking and Stability of the Arabidopsis IRON-REGULATED TRANSPORTER1. *The Plant Cell* **26**, 1294.

Kanwar P., Altmeisch S. & Bauer P. (2025) Quantitative tools for analyzing rhizosphere pH dynamics: localized and integrated approaches. *Biology Methods and Protocols* **10**.

Le C.T.T., Brumbarova T., Ivanov R., Stoof C., Weber E., Mohrbacher J., … Bauer P. (2016) Zinc finger of arabidopsis thaliana12 (zat12) interacts with fer-like iron deficiency-induced transcription factor (FIT) linking iron deficiency and oxidative stress responses1[OPEN]. *Plant Physiology* **170**, 540–557.

Meiser J., Lingam S. & Bauer P. (2011) Posttranslational Regulation of the Iron Deficiency Basic Helix-Loop-Helix Transcription Factor FIT Is Affected by Iron and Nitric Oxide. *Plant Physiology* **157**, 2154–2166.

Naranjo-Arcos M.A., Maurer F., Meiser J., Pateyron S., Fink-Straube C. & Bauer P. (2017) Dissection of iron signaling and iron accumulation by overexpression of subgroup Ib bHLH039 protein. *Scientific Reports* **7**, 10911.

Schenk S. & Schikora A. (2015) Staining of Callose Depositions in Root and Leaf Tissues. *BIO-PROTOCOL* **5**.
